# Antihypertensive and Antioxidant Activity of Chia Protein Techno-Functional Extensive Hydrolysates

**DOI:** 10.3390/foods10102297

**Published:** 2021-09-28

**Authors:** Alvaro Villanueva-Lazo, Sergio Montserrat-de la Paz, Noelia Maria Rodriguez-Martin, Francisco Millan, Cecilio Carrera, Justo Javier Pedroche, Maria del Carmen Millan-Linares

**Affiliations:** 1 Plant Protein Group, Department of Food and Health, Instituto de la Grasa—CSIC, Campus Universitario Pablo de Olavide, Edificio 46, Carretera de Utrera Km. 1, 41013 Seville, Spain; alvarovillanueva@ig.csic.es (A.V.-L.); noe91rm@gmail.com (N.M.R.-M.); fmillanr@ig.csic.es (F.M.); j.pedroche@csic.es (J.J.P.); 2 Department of Medical Biochemistry, Molecular Biology and Immunology, School of Medicine, Universidad de Sevilla, Avenida Dotor Fedriani 3, 41071 Seville, Spain; delapaz@us.es; 3 Department of Chemical Engineering, Universidad de Sevilla, Calle Profesor Garcia Gonzalez 1, 41012 Seville, Spain; cecilio@us.es; 4 Cell Biology Unit, Instituto de la Grasa—CSIC, Campus Universitario Pablo de Olavide, Edificio 46, Carretera de Utrera Km. 1, 41013 Seville, Spain

**Keywords:** chia protein hydrolysates, antihypertensive activity, antioxidant activity, functional food, techno-functional hydrolysate

## Abstract

Twelve high-quality chia protein hydrolysates (CPHs) were produced from chia protein isolate (CPI) in a pilot plant of vegetable proteins. To obtain functional hydrolysate, four CPHs were hydrolyzed by the action of Alcalase, an endoprotease, and the other eight CPHs were hydrolyzed by the action of Flavourzyme, an exoprotease. Alcalase-obtained CPHs showed significant antihypertensive properties particularly, the CPH obtained after 15 min of hydrolysis with Alcalase (CPH15A), which showed a 36.2% hydrolysis degree. In addition, CPH15A increased the antioxidant capacity compared to CPI. The CPH15A physicochemical composition was characterized and compared to chia defatted flour (CDF) and CPI, and its techno-functional properties were determined by in vitro experiments through the analysis of its oil absorption capacity, as well as the capacity and stability of foaming and emulsifying, resulting in an emulsifier and stabilizer better than the intact protein. Therefore, the present study revealed that CPH15A has potent antihypertensive and antioxidant properties and can constitute an effective alternative to other plant protein ingredients sources that are being used in the food industry.

## 1. Introduction

Considered as a superfood, chia (*Salvia hispanica* L.) is an oil seed that has burst into our diet with great impact due to its high nutritional and functional value [1]. Indeed, chia seeds contain high levels of fat (20% to 34%), particularly polyunsaturated fatty acids such as α-linolenic (60%) and linoleic (20%) acids. Its oil presents a low n−6/n−3 ratio, which improves the lipid metabolism and prevents the development of tumor cells, metabolic disorders and chronic diseases [2]. In addition to lipid composition, the levels of proteins reach 16% to 26%, prolamins being the main fraction, and the dietary fiber content ranges from 23% to 41% [3].

Recent research has evaluated the beneficial properties of bioactive compounds present in chia seeds, particularly on the immunological and hypolipemiant properties [2]. Moreover, the recent approval of chia seeds as a Novel Food by Regulation (EC) No 2015/2283 of the European Parliament has allowed chia consumption and incorporation in a wide range of foods. This authorization includes different food groups: bakery products, yoghurt, fruits, nut and seed mixes, breakfast cereals, juices, prepackaged chia seeds as such, fruit spreads, sterilized ready-to-eat meals based on cereal grains, pseudocereals and pulses [4].

Currently, health-conscious consumers demand a significant increase of plant proteins consumption compared to those from animals and, consequently, an overproduction of plant proteins [5]. In line with this trend, chia is an important source of high-quality protein (19–27%), greater than traditional foods such as rice (5.95%), wheat (9.61%) and oats (13.15%) [2] and similar to other seeds used for the generation of protein isolates such as hemp [6], peas, and chickpeas [5]. Protein isolates are high-quality food ingredients with protein contents close to 90%, which can improve both the nutritional composition and techno-functional characteristics of foods. Furthermore, a recent study showed that chia protein fractions possess functional properties for improving formulations in the food industry, opening new fields of study on the applicability of chia protein isolates (CPI) in the food industry [7]. However, protein isolates have two major limitations for their application in manufactured foods, such as their low solubility and the potential allergenicity of some components.

Taken together with the demand for new foods, many research groups have led the development of plant and animal protein hydrolysis processes in a wide range of raw materials, such as fish [8], asparagus [9], hemp [6,10], meat byproduct [11] and wheat gluten protein [12], among others. However, there are few studies that show the potential techno-functional uses of chia seed in the food industry and its application as a food ingredient containing peptides with biological activity. Thus, the aim of this work was to evaluate the suitability of the proteins of an emerging seed, such as chia proteins, for obtaining protein isolates and hydrolysates with bioactive and functional properties, which could be used as ingredients in the food industry for obtaining nutritious, healthy and functional foods.

## 2. Materials and Methods

### 2.1. Materials

*Salvia hispanica* L. seeds were supplied by the Autonomous University of Nuevo Leon (Monterey, Mexico). The enzymes used were Alcalase 2.4 L and Flavourzyme 1000 L, which were supplied by Novozymes (Novozymes, Bagsvaerd, Denmark). Thermostable α-amylase propylene glycol, 1-M glucose solution of amyloglucosidase, 2,4,6-trinitrobenzenesulfonic acid (TNBS), butylated hydroxytoluene (BHT), cholesterol and 2,2-diphenyl-1-picrylhydrazyl (DPPH) were purchased from Sigma Chemical Co. (Sigma Chemical Co., St Louis, MO, USA). All other chemicals were of analytical grade and purchased from Sigma Chemical Co. (St Louis, MO, USA) and Merk (Merck, Darmstadt, Germany).

### 2.2. Methods

The protein levels were determined by an elemental analysis using a LECO macro CN828 analyzer (LECO, St. Joseph, MI, USA) with a nitrogen-to-protein conversion factor of 6.25. The total dietary fiber was evaluated by the enzymatic gravimetric method [13]. The ash level was determined by the direct ignition method (550 °C for 36 h). Soluble sugars and polyphenols were analyzed using the standard curves of glucose and chlorogenic acid, respectively [14,15].

#### 2.2.1. Analysis of Amino Acid Composition by Ultra-High-Performance Liquid Chromatography (UHPLC)

The amino acid content was evaluated according to the method of Alaiz et al. with slight modifications [16]. The samples were hydrolyzed with HCl at 110 °C for 24 h in tubes sealed under nitrogen. Determination of the amino acid content in the acid hydro-lysate was carried out by ultra-high-performance liquid chromatography in a Acquity Arc equipped with a 2998PDA Detector, a Sample Manager FTN-R and a Quaternary Solvent Manager-R (Acquity Arc, Waters corporation, Milford, MA, USA), after derivatization with diethyl ethoxymethylenemalonate, using D,L-α-aminobutiric acid as the internal standard, and a 3 mm × 150 mm reversed-phase column (XSlect^®^ HSS T3, 2.5 µm) (Waters corporation, Milford, MA, USA). Two solvents: (A) 25-mM sodium acetate and 0.02% sodium azide (pH 6.0) and (B) acetonitrile were used to constitute a binary system gradient. For each amino acid, calibration curves were developed using a mix acid standard at the same hydrolysis conditions of the samples (Merck, Madrid, Spain), and the resultant peaks were detected by measuring its absorbance at 280 nm analyzed with EMPOWER software (Waters, Santa Clara, CA, USA). We used the Yust method to determine the tryptophan content [17].

#### 2.2.2. Molecular Weights (MWs) by Fast Performance Liquid Chromatography (FPLC)

MWs were estimated by gel filtration chromatography on an FPLC Akta Purifier 10 (GE Healthcare Bio-sciences AB, Uppsala, Sweden) equipped with a separation range of 1–300-kDa column Superose 12 10/300 GL. The standard proteins (GE Healthcare, Chalfont Saint Giles, UK) were used to calibrate the column: blue dextran (2000 kDa), aldolase (158 kDa), conalbumin (75 kDa), bovine serum albumin (67 kDa), ribonuclease A (13.7 kDa) and bacitracin (1.423 kDa). A calibration line was made using the logarithms of the MWs of these standard proteins and their elution volumes. For the elution, 50 mL of 0.05-M sodium phosphate buffer, 0.5-M sodium chloride and 0.02% (*w*/*v*) sodium azide adjusted at pH 7.5 with a flow of 1 mL/min was used. The concentration and injected volume of the samples were 30 mg/mL of protein and 500 μL, respectively. Protein elution was recorded by measuring its absorbance at 280 nm.

#### 2.2.3. Determination of Isoelectric Point of Chia Protein

To obtain a protein isolate, it is necessary to precipitate the previously extracted proteins, and the pH of minimum solubility (isoelectric point) of the chia proteins was determined according to Salcedo-Chávez et al., with some modifications [18]. The samples (2 g) were dissolved in 100 mL of H_2_O, and by adding NaOH or HCl, the pH was successively adjusted to 12, 10, 8, 6, 4 and 2, taking at each pH point an aliquot in duplicate, whose supernatant, obtained after centrifugation for 15 min at 9500× *g*, was measured in the nitrogen content by an elemental analysis, using a LECO macro CN828 analyzer (St. Joseph, MI, USA). A nitrogen-to-protein conversion factor of 6.25 was used.

#### 2.2.4. Generation of CPI

CPI was obtained at a pilot scale from 4 kg of Chia Defatted Flour (CDF), previously defatted in an Armfield FT29 extractor (Armfield Ltd., Hampshire, England), according to Lqari et al. [19]. The extraction ratio was adjusted to 1:40 (*w*/*v*) with 0.25% Na_2_SO_3_ (*w*/*v*) at a controlled pH of 10.5 for 1 h at ambient temperature under continuous stirring. After centrifugation in a decanter Tricanter Z23-3/441, (FLOTTWEG S.E., Vilsbiburg, Germany), the supernatant collected was centrifuged in a vertical centrifuge (Clara 20, ALFA LAVAL Iberia S.A., Madrid, Spain) to eliminate the residual solid particles and adjusted to pH 4.0 (isoelectric point of chia proteins) before being centrifuged again in the Tricanter Z23-3/441. Finally, the precipitated proteins were dried in a concurrent spray dryer PRODUCTION MINOR ^TM^ (GEA Niro, Søborg, Denmark) equipped with a 0.1-m diameter rotary wheel atomizer, at inlet temperature 190 °C, outlet temperature 90 °C, air volume flow of 250 m^3^/h and stored at room temperature.

#### 2.2.5. Generation of Chia Protein Hydrolysates

A CPI-distilled water mixture was made in a ratio of 7.5% *w*/*v* in a bioreactor that had temperature and pH control, and two types of hydrolysis were carried out with Alcalase 2.4 L, a serine endo-peptidase (2.4 Anson Unit (AU) g^−1^) and Flavourzyme 1000 L, a leucine amino-peptidase, which were kindly donated by Novozymes (Novozymes, Bagsvaerd, Denmark). The following conditions were used: CPI was hydrolyzed with Alcalase for 1 h at pH 8 and 50 °C, according to the manufacturer’s recommendations and Enzyme/Substrate relation (E/S) = 0.3 AU g^−1^ protein. On the other hand, CPI was hydrolyzed with Flavourzyme for 2 h at pH 7 and 50 °C, according to the manufacturer’s recommendations, and E/S = 50 Leucine Aminopeptidase Unit (LAPU) g^−1^ of protein. Every 15 min, an aliquot was taken, and to inactivate the enzyme, each aliquot was heated at 85 °C for 15 min. To obtain chia protein hydrolysates (CPHs), the samples were centrifuged at 9500× *g* for 15 min and the supernatants collected. The CPHs obtained using Alcalase were designated CPH15A, CPH30A, CPH45A and CPH60A, and those obtained using Flavourzyme were designated CPH15F, CPH30F, CPH60F, CPH75F, CPH90F, CPH105F and CPH120F, where the numbers indicated the time of hydrolysis in minutes.

#### 2.2.6. Determination of the Hydrolysis Degree (HD)

The TNBS method [20] was used to measure the HD, defined as the percentage of peptide bonds cleaved. To determinate the total number of amino groups in the sample, said sample is hydrolyzed with 6-N HCl for 24 h.

#### 2.2.7. Determination of the Angiotensin-Converting Enzyme (ACE) Inhibitory Activity

The ACE inhibitory activity was determined according to Sentandreu and Toldra with slight modifications [21]. Briefly, this method is based on the hydrolysis of the Abz-Gly-Phe (NO_2_)-Pro substrate by the action of ACE that gives rise to the fluorescent product o-aminobenzoylglycine. The Abz-Gly-Phe (NO_2_)-Pro substrate was dissolved in 0.15-M tris buffer and 1.125-M NaCl, pH 8.3 to obtain a final 0.45-µM substrate concentration and maintained at 4 °C until use. The ACE previously dissolved in 50% glycerol at a concentration of 1 unit/mL was diluted in 0.15-M tris buffer containing 60-µM ZnCl_2_ at pH 8.3, the final enzyme concentration being 0.04 unit/mL. A multi-well black polystyrene plate (Porvair Filtration Group, Leatherhead, UK) was used, where 40 µL of tris buffer for the targets or ACE solution was added to each well. To account for the interference of the compounds, a sample blank was prepared, which was treated in the same manner using distilled water instead of the sample. The enzymatic reaction was started by adding 160 µL of the fluorescent substrate that was immediately mixed and incubated at 37 °C in the fluorimeter. The generated fluorescence was measured after 60 min using as excitation and emission wavelengths 350 and 420 nm, respectively. FLUOstar Control version 1.32 R2 software was used to process the data. The inhibitory activity of each sample was determined in triplicate, and the following formula was used for its calculation:

ACE inhibitory activity (%): ((Fc-Fb) − (Fs-Fsb))/(Fc-Fb)

Fc: control fluorescence; Fb: blank fluorescence; Fs: sample fluorescence; Fsb: sample blank fluorescence.

The IC50 value was defined as the concentration of hydrolysate in µg protein/mL required to produce 50% inhibition of ACE in the conditions described above and determined by a regression analysis of ACE inhibition (%) versus the protein hydrolysate concentration.

#### 2.2.8. Determination of Antioxidant Activity

##### Determination of the Antioxidant Activity by β-Carotene-Linoleic Acid Assay

CPI and CPHs’ properties to prevent the bleaching of β-carotene were determined by Marco [22]. In brief, 10 mL of chloroform were added to two milligrams of β-carotene; after that, 200 mg of tween 20, 20 mg of linoleic acid and a aliquot of 1-mL β-carotene solution were mixed, and the chloroform was removed under vacuum in a rotary evaporator. The resulting mixture was vigorously stirred after adding 50 mL of distilled water enriched in oxygen. A small amount (0.1 mL) of each sample (at 10 mg/mL of protein) and 2.5 mL of the β-carotene–linoleic acid emulsion were mixed in test tubes. The absorbance at 470 nm was immediately recorded, which was considered as t = 0 min, against a blank containing β-carotene–linoleic acid emulsion without a β-carotene reagent and BHT (at 0.08 mg/mL) as the positive control. The absorbance was again measured after incubation at 50 °C for 60 min. The equation IP = (A/A_0_) × 100 was used to calculate the index protection (IP), where A is absorbance at 60 min and A_0_ is the absorbance at time zero.

##### Ferric Reducing Antioxidant Activity

The capacity of the CPI and CPHs to reduce iron (III) was determined as described by Oyaizu [23]. A mixture of 0.25 mL of 0.03-mol/L potassium ferricyanide, 0.25 mL of 0.2-mol/L sodium phosphate buffer, pH 6.6 and 0.1 mL of each sample (10 mg/mL of protein) were made and were incubated at 50 °C for 20 min. Then, 0.25 mL of 0.6-mol/L trichloroacetic acid was added, and the mixture was centrifuged at 1300× *g* for 10 min. Five hundred microliters of supernatant were mixed with 0.1 mL of 3.7-mmol/L ferric chloride and 0.5 mL of distilled water. The absorbance of each resulting solution was measured at 700 nm after 10 min. An increased absorbance of the reaction mixture indicated an increased reducing power. The reducing power of each sample was measured in triplicate, and BHT at 0.08 mg/mL was used as the positive standard.

##### DPPH Radical Scavenging Power of Protein Products

According to Wu [24], a synthetic free radical compound (DPPH) can be used to analyze the ability of CPI and CPHs to scavenge free radicals. An aliquot of 1.5 mL of each sample (10 mg/mL of protein) was transferred to a test tube and mixed with 0.1-mmol/L DPPH in ethanol. The mixture was shaken and kept in the dark for 30 min at ambient temperature before measuring its absorbance at 517 nm, where DPPH has an absorption band that disappears upon reduction by an antiradical compound. BHT (0.08 mg/mL) was used as the positive standard and distilled water as the control, both being conducted in the same manner of sample. All the samples were measured in triplicate. The inhibition percentage was expressed as [(A_control_ − A_sample_)/A_control_] × 100, where A_sample_ is the absorbance of the sample, and A_control_ is the absorbance of the control reaction.

#### 2.2.9. Functional Properties

##### Solubility

To study the solubility of Chia protein, the method of Salcedo-Chávez et al. with slight modifications [18] was used. The samples (2 g) were dissolved in 100 mL of H_2_O, and by adding NaOH or HCl, the pH was successively adjusted to 12, 10, 8, 6, 4 and 2, taking at each pH point an aliquot in duplicate whose supernatant, obtained after centrifugation for 15 min at 9500× *g*, was measured in the nitrogen content by an elemental analysis, with a nitrogen-to-protein conversion factor of 6.25, using a LECO macro CN828 analyzer (LECO, St. Joseph, MI, USA). The solubility was calculated as the percentage of the total nitrogen of the original sample present in the soluble fraction.

##### Oil Absorption

Oil absorption was determined according to Lin et al. [25]. To express oil absorption, we measured the grams of oil that 100 g of the sample were able to retain.

##### Foaming Capacity and Stability

The method of Fuhrmeister and Meuser was used with slight modifications [26]. Fifty milliliters of 3% (*w*/*v*) sample dispersion in distilled water were homogenized in a model Omnimixer homogenizer (Sorvall Instruments, Wilmington, DE, USA) at rpm. The foaming capacity was calculated as a percent increase in the foam volume, and the foam stability was determined by measuring the foam capacity after 60 min at room temperature.

##### Emulsifying Activity and Stability

The emulsifying activity and stability were analyzed according to Bejosano and Corke with slight modifications [27]. Briefly, 50 mL of 5% (*w*/*v*) sample suspension were homogenized in a Omnimixer homogenizer (Sorvall Instruments, Wilmington, DE, USA) at rpm for 30 s. This was followed by a first addition of corn oil (25 mL) and subsequent homogenization for 30 s; after which, a second addition of corn oil (25 mL) and subsequent homogenization was carried out, this time for 90 s. The emulsion was centrifuged at 1100× *g* for 5 min. To calculate the emulsifying activity, the volume of the emulsified layer was divided by the volume of emulsion before centrifugation. To calculate the emulsifying stability, emulsions prepared by the above procedure were heated for 15 min at 85 °C, cooled to room temperature and centrifugated at 1100× *g* for 5 min again. The emulsion stability was expressed as the percentage of emulsifying activity remaining after heating.

#### 2.2.10. Statistical Analysis

All values were expressed as the arithmetic means ± standard deviations (SD). The data were evaluated with Graph Pad Prism Version 5.01 software (GraphPad Software Inc., San Diego, CA, USA). Group-wise statistical comparisons were performed by a one-way ANOVA with a post hoc Bonferroni test. The differences were considered to be significant at *p* < 0.05.

## 3. Results and Discussion

### 3.1. Generation of Chia Protein Isolate

To obtain CDF, chia seeds were defatted with hexane at the pilot scale. CDF showed a protein content of 34.95% similar to soybean flour protein, worldwide used in animal and human feeding [28] and was used to obtain CPI for further analysis and enzymatic hydrolysis. Indeed, the minimum solubility pH of chia proteins was determined in pH 4.0, in order to optimize the production of CPI, which showed a protein content of 82.85%, a yield by weight of 19.7% and a protein recovery of 46.4%. Similar results have been obtained for protein isolates of other emerging crops [29].

### 3.2. Generation of Chia Protein Hydrolysates

**Figure 1:**
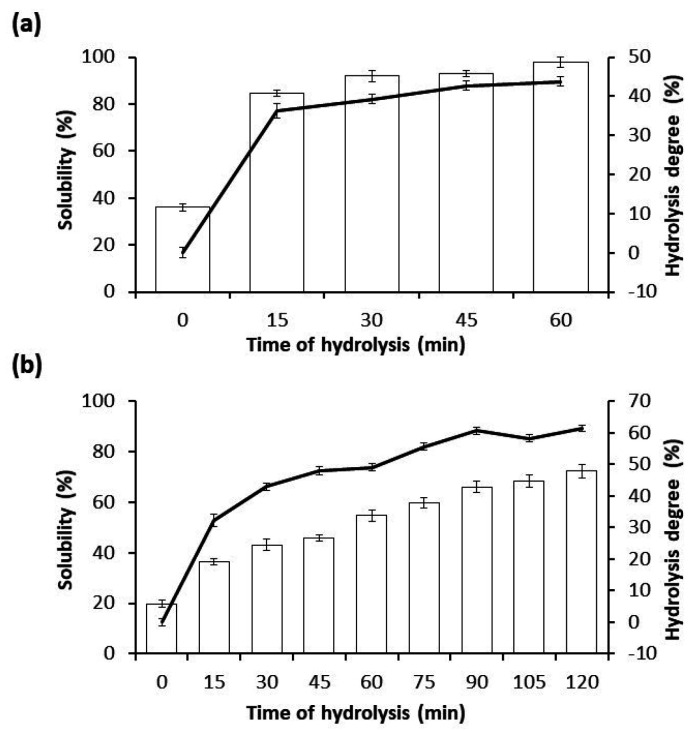
Time course of the hydrolysis degree (solid curve) of CPI during enzymatic hydrolysis with Alcalase (**a**) at 0, 15, 30, 45 and 60 min and with Flavourzyme (**b**) at 0, 15, 30, 45, 60, 75, 90, 105 and 120 min. Additionally, both bar graphs express the % of solubility for each case. Data is ex-pressed as the mean ± standard deviation of at least three determinations.

CPI was used to assay two types of hydrolysis: one with Alcalase 2.4 L, an endoprotease (Figure 1a), and other one with Flavourzyme 1000 L, an exoprotease (Figure 1b), resulting in four CPHs using Alcalase, designated as CPH15A, CPH30A, CPH45A and CPH60A, and eight CPHs using Flavourzyme, designated as, CPH15F, CPH30F, CPH45F, CPH60F, CPH75F, CPH90F, CPH105F and CPH120F. In both cases, an initial sample was taken, before adding the enzyme, which was treated the same as the other samples. Alcalase and Flavourzyme are extensively used in the food industry, since their optimal hydrolytic activity is found in a mild pH range (7–10) and their temperature between 50 and 70 °C, which is great interest to avoid contamination in the process by preventing bacterial growth. On the other hand, both enzymes are totally soluble in water, complying with the specifications recommended by the FAO (Food and Agriculture Organization of the United Nations) [30] and JECFA (Joint Committee of Experts on Food Additives) [31] related to enzymes for food use. During Alcalase-mediated hydrolysis reaction, a high hydrolysis ratio was showed at the first 15 min (CPH15A), due to its endoprotease action, reaching a HD of 36.2% and a solubility of 84.7%. After that time, the HD of CPHs did not show significant variations, reaching at the end of hydrolysis reaction (CPH60A) a HD of 43.8% and a solubility of 97.7%. A high solubility value is important to allow the use of CPHs in liquid formulations, such as juices or fortified beverages [32]. During a Flavourzyme-mediated hydrolysis reaction, the HD showed a constant increase of the hydrolysis, reaching a HD of 32.3% at 15 min (CPH15F), of 49.07% at 60 min (CPH60F) and of 61.4% at 120 min (CPH120F). Flavourzyme is an exoprotease that produces the release of individual amino acids, which involves a slight but constant increase of HD and protein solubility. Similar results are observed for hydrolysates obtained from quinoa and wheat protein isolates [11,33].

### 3.3. ACE Inhibitory Activity of Chia Protein Hydrolysates

ACE activity produces an increase in blood pressure due to the production of angiotensin-II, a vasoconstrictor peptide, and to the degradation of bradykine, a vasodilator peptide. The use of ACE inhibitors (enalapril, captopril, lisinopril and benazepril, among others) is widespread in pharmacotherapy against hypertension [34]. The scientific literature has shown a wide variety of animal and plant protein products with ACE inhibitory activity [35]. As shown in Table 1, CPHs obtained Alcalase and induced an ACE inhibitory activity about 80% at the concentration of 200 μg/mL. Segura-Campos et al. (2013) [36] showed that the addition of the CPHs at three hydrolysis times (90, 120 and 150 min) to white bread resulted in products with higher ACE inhibitory activity than the control treatment. The CPHs showed a higher ACE inhibitory activity compared to those obtained by Wheat Gluten Protein Hydrolysates [11] and others ACE-inhibiting peptides obtaining by enzymatic hydrolysis of plants proteins such as soy, rice or peas [37].

However, no significant differences were observed among the hydrolysates obtained with the same enzyme, and it was not possible to correlate the inhibition of ACE with the time of hydrolysis. The CPHs obtained using Flavourzyme showed a greater inhibition of ACE when compared to those produced by CPI, although the ACE inhibitory values (less than 40%) were lower than those obtained using Alcalase at the same concentration. Once again, no direct correlation was observed between the ACE inhibition and hydrolysis time. These results are similar to those regarding the antioxidant, ACE inhibitory and antiproliferative activity of Cicer protein hydrolysates [38].

Taking together with the previous results, the CPHs obtained using Flavourzyme were discarded for the following analyses, and the CPHs obtained using Alcalase were tested at a concentration range to calculate the concentration required to produce 50% of the ACE inhibition (IC50). The results showed that the four CPH selected had an IC50 in the 67.50–78.84 range in µg/mL. Compared to other chia family members, such as basil, a good ACE inhibitory activity in a dose-dependent manner was reported [39]. In addition, different peptides with ACE inhibitory activity were also identified in lemon basil seeds [40]. Interestingly, chia seeds showed higher ACE inhibitory activity than those founded in basil leaves and seeds. Indeed, the use of CPHs may improve the ACE inhibitory activity observed in gluten-free layer cakes with 10–20% of raw chia seed flour supplementation [41].

### 3.4. Antioxidant Activity of Chia Protein Hydrolysates

**Figure 2:**
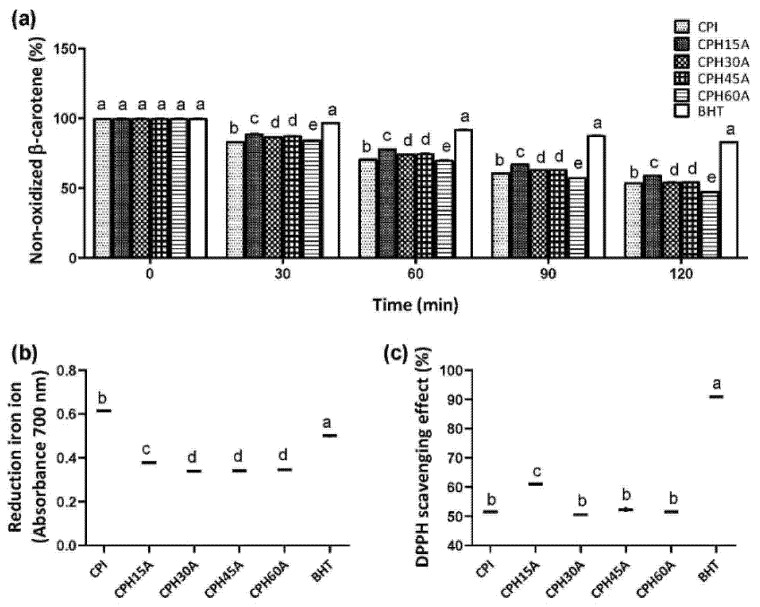
Nonoxidized β-carotene (**a**), ferric reductor antioxidant power (FRAP) (**b**) and DPPH radical scavenging activity (**c**) by CPI and CPHs obtained using Alcalase in cell-free in vitro experiments. Values are represented as the means ± standard deviation of three determinations. Statistical differences are marked with different letters (*p* < 0.05).

The antioxidant activity of protein hydrolysates may be due to several mechanisms [42], so three assays were used: decolorization of β-carotene (Figure 2a), measure of ferric reducing antioxidant power (Figure 2b) and measure of capacity to sequester the DPPH free radical (Figure 2c). BHT was always used as a positive control in all the experiments. According to the ACE results, the CPHs obtained using Alcalase were selected to carry out the antioxidant assays. The results showed that only CPH15A had a higher antioxidant activity compared to those obtained: CPH30A, CPH45A and CPH60A. Regarding the ferric-reducing antioxidant power, CPI showed the higher antioxidant activity, similar to BHT, probably due to the presence of other nonprotein compounds with antioxidant activity. In line with our results, Silveira Coelho et al. (2019) [43] showed that chia protein concentrates hydrolyzed with Flavourzyme, Alcalase and a sequential system had a potential antioxidant capacity.

### 3.5. Chemical Characterization of Chia Protein Products

Chia protein products such as CDF, CPI and CPH15A were chemically characterized. As showed in Table 2, after extracting the oil, CDF presented a protein content of 34.95%.

A high protein composition is a basic value to use this byproduct to obtain protein concentrates, isolates and hydrolysates. The procedure to obtain a protein isolate usually involves protein extraction and the elimination of the other components. However, in CDF, it was not possible to eliminate the fiber content, which represents 11.01% in CPI and remains in CPH15A. Probably, this fiber must be a soluble dietary fiber from the mucilage, which confer good foaming and emulsifying properties [44], and would be incorporated into the hydrolysate. Indeed, to evaluate the foaming and emulsifying properties would be interesting in CPHs. Finally, CPH15A showed a slightly lower protein content compared to those found in CPI as a result of the ash increase caused by the addition of sodium hydroxide to maintain the optimal pH for the enzymatic reaction.

### 3.6. Amino Acid Composition of Chia Protein Products

Amino acid composition of the chia protein products (Table 3) broadly complies with the adult requirements established by the Food and Agriculture Organization/World Health Organization/United Nations University (FAO/WHO/UNU) for indispensable dietary amino acid [45].

**Figure 3:**
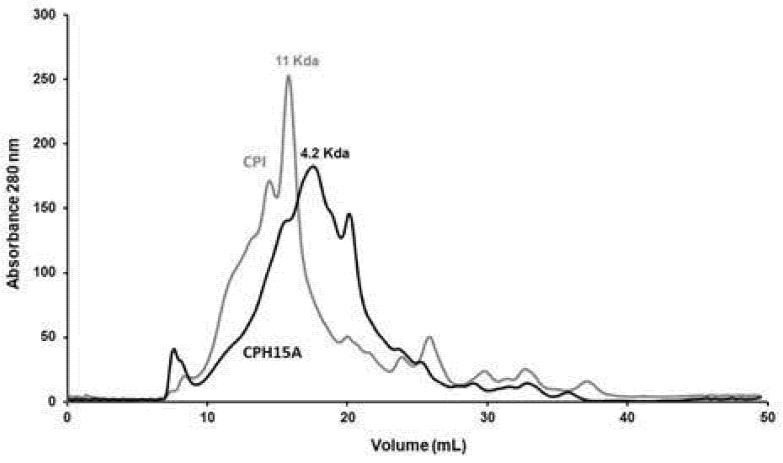
Molecular weight profiles by size exclusion FPLC of CPI and CPH15A.

The predominant essential amino acids were sulfur amino acids (methionine and cysteine) and aromatic amino acids (phenylalanine, tyrosine and tryptophan). In this last group, the tryptophan levels of the protein products were those that showed the greatest differences with respect to the requirements established in the standard, relevant since sulfur-containing amino acids represent a powerful part of the cell antioxidant system [46]. Broadly, no significant differences were observed between CDF, CPI and CPH15A, which indicates that the amino acids were not damaged during the enzyme process. Figure 3 showed how hydrolysis with Alcalase reduced the molecular weight of the majority of the CPI proteins (11 Kda), which became 4.2 kDa in CPH15A. It has been reported that the peptides that have the highest biological activity are those of low molecular weight (2–20 amino acids) [47].

### 3.7. Functional Properties

Foods containing protein hydrolysates can improve their nutritional and functional properties [41,48]. Plant protein hydrolysates used in food can be divided into two large groups according to their HD. One of them, limited hydrolysates, with a HD between 1 and 10%, are used for the improvement of the functional properties, such as solubility, emulsifying, foaming capacity and absorption of water and oil [49]. The second one, extensive hydrolysates with a HD higher than 10%, are used for the generation of specialty food, mainly in the preparation of hypoallergenic and bioactive foods. CPH15A showed a HD of 36.2%; therefore it was an extensive hydrolysate that was not expected to have functional properties, since, in general, there are limited hydrolysates that present better functional properties [50]. However, as mentioned above, CPH15A had the particularity of containing a high content of soluble fibers from mucilage, which has water retention and emulsifier and stabilizer properties [51], hence the interest in studying the functional properties of CPH15A.

**Figure 4:**
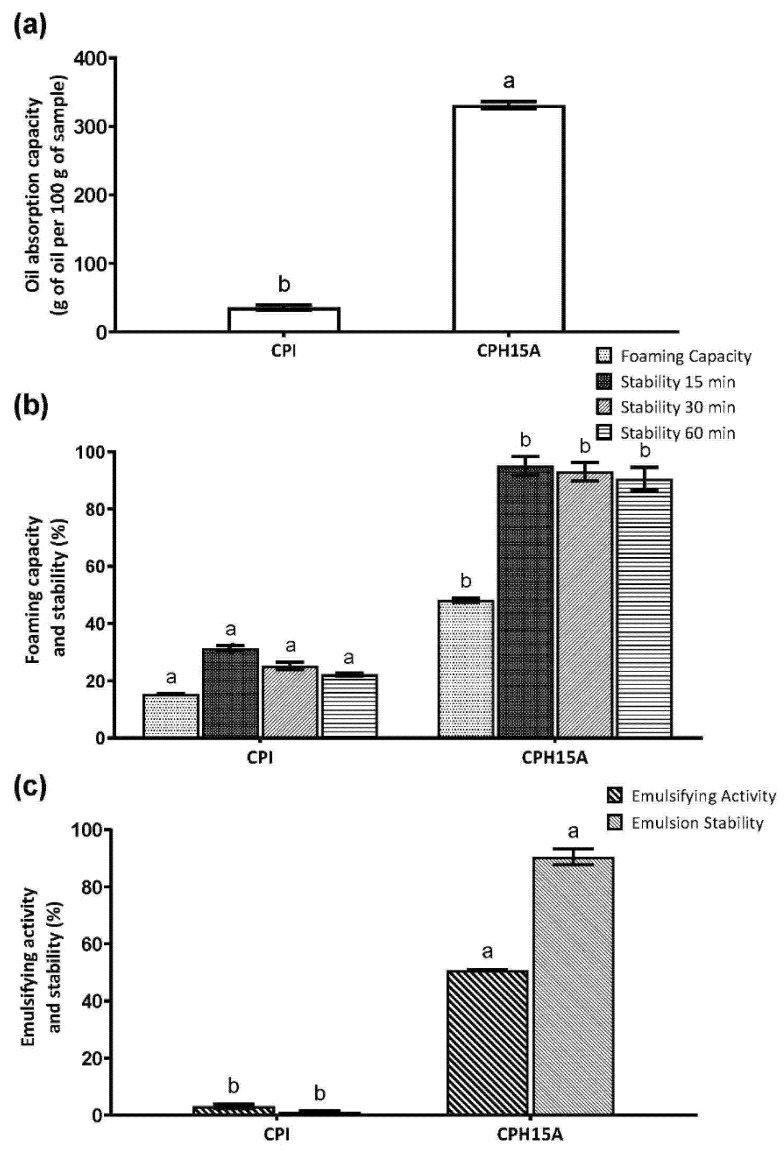
Oil absorption capacity (**a**), foaming capacity and stability (**b**), and emulsifying activity and stability (**c**) by CPI and CPH15A obtained using Alcalase. Values are represented as the means ± standard deviation of three determinations. Statistical differences are marked with different letters (*p* < 0.05).

CPH15A increased the oil absorption capacity by more than 200 times compared to CPI (Figure 4a). The oil absorption capacity may be defined as the ability to adhere fats in a physical entrapment of oil, and it may impact on the emulsifying capacity [52].

The foaming properties of proteins are important to predict their functionality in aerated foods. As depicted in Figure 4b, the foaming capacity of CPH15A was higher than CPI; on the other hand, it was similar to other protein hydrolysates such as chickpeas [50].

Both protein sources and their structures, molecular weights and adsorption behaviors influence the emulsifying properties of food [53]. An increase in emulsifying activity due to protein hydrolysis has been described for corn, lupin soy, rice bran, pumpkin, oat, sesame, chickpea, arachin, rapeseed and potato samples [54]. The emulsifying capacity of CPI and CPH15A is shown in Figure 4c. CPH15A showed an emulsifying capacity 50 times higher and 100 times more stable than the intact proteins. There is currently a lot of research underway on the functional properties of plant proteins that may be an alternative to dominant natural food emulsifiers, dairy and egg proteins [55].

## 4. Conclusions

CPHs obtained using Alcalase showed antihypertensive properties with more than 80% of the ACE inhibitory activity and an IC50 less than 80 µg/mL. In addition, CPHs obtained using Alcalase showed antioxidant properties by the decolorization of β-carotene, measure of ferric-reducing antioxidant power and measure of the capacity to sequester DPPH free radicals. Due to its high content of protein and dietary soluble fiber, CPH15A with a hydrolysis degree of 36.2% showed a similar emulsifying capacity and a higher foaming capacity and emulsifying stability that those produced by others limited the protein hydrolysate with a lower hydrolysis degree, which is generally admitted to having functional properties. In conclusion, the results of this study indicate the potential use of CPH15A in the production of functional foods. However, further in vivo studies should be performed to elucidate the antihypertensive and antioxidant activities of CPHs.

## Figures and Tables

**Table 1:** ACE inhibitory activity (%) of CPI hydrolyzed with Flavourzyme or Alcalase at different times. Maximal inhibitory activity (IC50) of CPHs obtained using Alcalase on the ACE enzyme.

Hydrolysis Time (min)	CPH Flavourzyme	CPH Alcalase	
	ACE Inhibition (%)	ACE Inhibition (%)	IC_50_ (µg/mL)
0	15.92 ± 0.27	12.33 ± 0.54	
15	42.19 ± 0.53	82.85 ± 0.42	78.84 ± 1.21
30	36.17 ± 0.83	80.13 ± 0.40	74.63 ± 0.53
45	40.50 ± 0.40	82.16 ± 0.25	67.50 ± 0.44
60	36.66 ± 0.62	82.84 ± 0.13	68.06 ± 0.67
75	28.79 ± 0.74		
90	34.33 ± 0.84		
105	30.36 ± 0.61		
120	31.83 ± 2.04		

**Table 2:** Chemical composition (g/100 g) of chia protein products. CDF, chia defatted flour; CPI, chia protein isolate; CPH15A, chia protein hydrolysate obtained after15 min of hydrolysis with Alcalase.

	CDF	CPI	CPH15A
Moisture	7.86 ± 0.07	4.80 ± 0.30	7.32 ± 0.08
Ash	6.72 ± 0.05	0.13 ± 0.09	6.45 ± 0.17
Proteins	34.95 ± 0.43	82.85 ± 0.11	75.03 ± 0.45
Fiber	50.46 ± 1.98	11.01 ± 0.80	11.20 ± 0.70

**Table 3:** Summary of the adult indispensable amino acid composition and requirements of chia protein products. CDF, chia defatted flour; CPI, chia protein isolate; CPH15A, chia protein hydrolysate obtained after 15 min of hydrolysis with Alcalase.

Amino Acid Protein	CDF	CPI	CPH 15A	2007 FAO/WHO/UNU ^a,b^
Histidine	43.9 ± 1.6	37.9 ± 2.9	39.2 ± 0.5	15
Isoleucine	32.9 ± 0.8	35.2 ± 0.3	35.1 ± 0.0	30
Leucine	67.5 ± 0.7	70.4 ± 4.6	72.2 ± 0.7	59
Lysine	48.0 ± 1.1	46.4 ± 1.4	48.1 ± 0.6	45
Methionine + cysteine	44.9 ± 0.9	40.5 ± 1.9	40.3 ± 1.2	22
Methionine	24.4 ± 0.5	21.9 ± 1.8	21.1 ± 2.1	16
Cysteine	20.5 ± 1.3	18.6 ± 1.9	19.2 ± 0.4	6
Phenylalanine + tyrosine	147.3 ± 0.2	136.7 ± 1.9	135.5 ± 1.1	38
Threonine	37.2 ± 1.1	38.4 ± 2.1	38.0 ± 0.3	23
Tryptophan	47.3 ± 0.7	48.1 ± 0.6	49.3 ± 0.3	6
Valine	44.8 ± 0.7	46.9 ± 0.9	47.6 ± 0.8	39
Total indispensable aminoacids	513.3	500.5	505.3	277

^a^ FAO/WHO/UNU. Scoring pattern mg/g protein requeriment in adults. ^b^ FAO and FINUT 2017. Dietary protein quality evaluation in human nutrition. FAO Food and Nutrition Paper NO. 92.

## Data Availability

The data is contained within this article.
